# Utilising clinical associates to address mental health service provision challenges in South Africa: the views of healthcare managers and providers

**DOI:** 10.1186/s12913-025-13300-9

**Published:** 2025-08-18

**Authors:** Saiendhra Vasudevan Moodley, Jacqueline Wolvaardt, Christoffel Grobler

**Affiliations:** School of Heath Systems and Public Health, University of Pretoria, Pretoria, South Africa

**Keywords:** Mental health, Mental illness, Psychiatry, Clinical associates, Mental health workforce, Task-sharing, Focus groups

## Abstract

**Background:**

A constraint in South Africa’s mental health system is the human resources required to provide services. Given the shortage of specialist mental health professionals, the use of non-specialists such as clinical associates in mental health task-sharing is essential. The study aimed to explore the views of health managers, doctors, and nurses in four districts of South Africa on the scale of mental illness seen in their health services, their human resources challenges, and their attitudes towards mental health task-sharing involving clinical associates.

**Methods:**

Focus group discussions were conducted in one district in each of the four provinces where clinical associates were known to be employed. Focus group participants were purposively sampled to ensure that each focus group consisted of a combination of managers and healthcare professionals from hospital and primary health care levels. Audio recordings were transcribed, and thematic analysis was conducted.

**Results:**

A total of 29 individuals participated. Four themes emerged from the focus group discussions. The first theme to emerge was ‘mental illness is not going away’ with substance use, increasing numbers of younger patients, and high rates of relapse and readmission accounting for this. The second theme identified was ‘the health system cannot cope with mental illness’ as mental health had not been prioritised and this had resulted in a lack of mental health units and beds, deficiencies at primary health care level, and human resources for mental health challenges. The third theme was ‘clinical associates could be part of the remedy’ based on past experience in other disciplines but constraints such as their scope of practice would need to be addressed. The final theme identified was ‘specialised clinical associates could help mend the mental health system’ but this would require a review of employment policies and a clarification of their roles.

**Conclusions:**

Mental health is a significant and expanding concern. Clinical associates could help alleviate human resource constraints in mental health with enhanced undergraduate and advanced training. However, overcoming structural barriers such as scope of practice, regulatory issues, and creation of posts will be crucial to realise their potential contribution.

**Supplementary Information:**

The online version contains supplementary material available at 10.1186/s12913-025-13300-9.

## Background

The mental health system in South Africa (SA) faces numerous challenges. Despite having mental health legislation and policy that is considered to be progressive and comprehensive, there has been a failure regarding implementation of this legislation and policy within SA’s already overburdened health system [1, 2]. Challenges related to governance include the low prioritisation of mental health, the lack of managerial and planning capacity at provincial and district level, and weak intersectoral collaboration [1]. More than 80% of SA’s population are uninsured [3] and rely on SA’s public healthcare sector for their health services including those for mental health [4]. Less than 5% of SA’s total health budget was spent on providing mental health services in the public sector in the 2016/17 financial year with wide disparities in per capita expenditure between the provinces [2]. Inpatient care accounted for 86% of mental healthcare expenditure in this period [2]. Modelling by Docrat et al. [2] suggests that a miniscule proportion of the uninsured population requiring mental healthcare actually received inpatient care (0.89%) or outpatient care (7.35%) in 2016/17. Significant mental health system challenges that have been identified include the lack of a dedicated mental health budget, infrastructure constraints, medication supply issues, and mental health workforce availability [1, 2].

A key constraint of SA’s mental health system is the human resources required to provide mental health services. van Rensburg et al. [5] reported a ratio of 1.53 psychiatrists per 100 000 population in 2019, with the majority of psychiatrists concentrated in urban centres. Access to specialist mental health professionals in rural areas in SA is a challenge [5–7]. Substantial disparities exist in access to psychiatrists between the private and public sector with Docrat et al. [2] estimating that there were only 0.31 psychiatrists in the public sector per 100 000 uninsured population in 2018 [2]. This ratio is significantly lower than the target of 1.03 psychiatrists per 100 000 population for SA suggested by Bruckner et al. [8]. This ratio was 0.15 or less in five of the nine provinces with only 0.08 psychiatrists in the public sector per 100 000 uninsured population in Mpumalanga [2]. The ratio of psychologists in the public sector per 100 000 uninsured population was 0.97 in 2018 [2]. These ratios for public sector occupational therapists and public sector social workers were 1.53 and 1.83 per 100 000 uninsured population respectively [8]. In addition to the numbers of personnel available to deliver mental health services, other human resources challenges that have been identified including the creation of appropriate posts, inadequate pre-service training in mental health for non-specialists, a high workload and high turnover of staff, and resistance and negative attitudes of non-specialists to treat mental health patients [1].

Given the shortage of specialist mental health professionals, the use of non-specialists in mental health task-sharing is essential. In addition to medical officers and registered nurses, clinical associates could potentially be utilised to deliver mental health services. Clinical associates are a mid-level medical worker that complete a three-year undergraduate degree named Bachelor of Medicine in Clinical Practice at Walter Sisulu University and the Bachelor of Clinical Medical Practice at the University of Pretoria and University of Witwatersrand [9].Similar cadres exist in a number of countries including Angola, Kenya, Malawi, Mozambique, Tanzania, Uganda, Zambia, Zimbabwe, Canada, Netherlands, United Kingdom and the United States of America [9–13]. The mid-level medical cadre are referred to by different names depending on the country including clinical officers, medical assistants, health officers, physician assistants, and physician associates [11, 13, 14]. The process to establish the clinical associate cadre in SA has been described in detail by Couper and Hugo [9] and included a national consultative process to determine their scope of practice, a review of models from the USA and other African countries, and the development of a common national curriculum document. Clinical associate training in SA is mainly district-hospital based with early clinical involvement [9]. The intention of the training is to prepare clinical associates to diagnose and manage common medical conditions at primary care and district hospital levels working under the supervision of a medical doctor [15]. There were more than 2250 clinical associates registered with the Health Professions Council of South Africa in July 2025 [16] with between 70 and 140 graduating annually from the three universities with training programmes [15].

The Regulations [17] governing clinical associates’ scope of practice includes tasks related to mental health which are included under their list of ‘procedures’ viz. mental health history, mental health examination, mini-mental state examination, family/mental health counselling. The three undergraduate training programmes in SA all include a mental component that ranges from two to four weeks in their final year of study [18]. Interviews with academics and clinicians involved in their training and class representatives found strong support for role for clinical associates in delivering mental health care [19]. There is evidence that a number of clinical associates are already engaged in mental health-related work in SA [20] as are similar cadres in other parts of Africa and in the United States of America [21–24]. The broader use of clinical associates in mental health service provision in SA would depend (amongst other factors) on the perceived current need for their involvement in the mental health system and acceptability of mental health task-sharing involving clinical associates to managers and clinicians involved in mental health. The study aimed to explore health managers’, medical doctors’ and registered nurses’ views in four districts of SA on the scale of mental illness in their health services, mental health human resources challenges faced, and their attitudes towards mental health task-sharing involving clinical associates.

## Methods

### Study design

An exploratory, qualitative study was conducted underpinned by a phenomenological methodological orientation.

### Study setting

Focus group discussions were conducted in four districts of South Africa (one per district) where clinical associates were known to be employed (Table 1).

**Table 1:** Participating districts

Province	District	Focus group duration (minutes)
Eastern Cape	Sarah Baartman	67:05
KwaZulu Natal	eThekwini*	89:50
Mpumalanga	Gert Sibande	58:17
North West	Dr Kenneth Kuanda	74:14

*a metropolitan municipality

### Study population and sampling

The study population were doctors and registered nurses involved in mental health service provision in the public sector as well as health managers in the study sites. Participating health managers were those that were responsible for mental health at district (or subdistrict) level and managers of hospitals, community health centres (CHCs), or primary health care (PHC) clinics. Participating registered nurses and doctors were those treating mental health patients at hospitals where clinical associates were employed. Participating registered nurses and doctors from CHCs or PHC clinics were drawn from those that offer mental health services but were not required to have clinical associates on their staff establishment. Focus group participants were purposively sampled to ensure that each focus group consisted of a combination of managers and clinicians as well as participants from hospital level and PHC facilities. The number of participants comprised between 6 and 8 participants [25].

### Measurement tool

A focus group discussion guide with open-ended questions (and probes) was developed for this study (Supplementary File 1). Focus group questions included the participants’ perceptions of the scale of the mental health problem in their district, their views on whether there were sufficient human resources to address it, and their views on whether clinical associates have a role to play in mental health task sharing. Test interviews were conducted prior to the focus groups with a clinician and hospital manager who were not part of the study to explore language and clarity of the questions [26].

### Data collection

Potential participants were identified in each of the selected districts with the assistance of a hospital manager and a district-based manager or clinician. They were contacted initially by e-mail or telephone (depending on what contact information was available) to brief them about the study. Further information including the informed consent document was e-mailed to those who indicated interest in participating. The focus groups took place between November 2022 and March 2023. Three of the focus groups were conducted in the boardroom or meeting room of the hospitals where the hospital-based participants worked. One of the focus groups was held in a private venue due to the long distance to the hospital for the non-hospital-based participants. Refreshments were provided prior to the start of each of focus group. The researcher (a public health medicine specialist based at the time in Gauteng province who had no prior relationship to the participants) acted as the focus-group facilitator. There was a different observer present at each of the four focus groups and they provided written or verbal feedback on their observations to the researcher following the focus group discussion. The focus groups commenced with a review and signing of the informed consent document and the interviewer taking the participants through the processes to be followed e.g. referring to each other by their allocated numbers rather than names during the interview. All of the individuals present at the briefing provided informed consent. With the exception of one participant at one of the focus groups who excused himself due to an urgent matter but later returned, all participants remained until the focus group discussions were complete. The duration of each focus group is shown in Table 1. Focus groups were recorded using two audio recording devices placed in different locations [27].

### Data management and analysis

The audio recordings were uploaded and stored on password-protected computer. Backup copies were stored in a password-protected cloud account. A professional transcription service was used to transcribe the focus group discussions. The transcripts were sent back to all the focus group participants for member checking. The transcripts were then imported into Atlas.ti version 23 for analysis. The analysis process began by reading through the text, memoing emergent ideas, and forming initial codes [27]. The codes were then grouped into categories [27]. The analysis produced 224 codes and 20 code groups. The coding was done by the first author with the second author reviewing the codes and code groups. Subthemes and themes were then generated.

### Processes to ensure quality of research

Techniques used to ensure the trustworthiness of the data included the presence of an observer at each focus group, ‘thick description’, peer debriefing, member checking, iterative data analysis, and maintaining an audit trail [28].

## Results

### Participant characteristics

There was a total of 29 participants in the focus groups across the four districts (randomly designated as A, B, C, and D). Just under of half of the participants (*n* = 14, 48.3%) in the four focus groups were between 40 and 49 years of age and just over half of participants (*n* = 15, 51.7%) were female (see Table 2). The majority of participants were registered nurses (*n* = 17, 58.6%) and 12 (41.4%) were medical doctors with three of these indicating they were medical specialists. A total of 13 participants (44.8%) had management roles with nine of these having purely managerial roles.

**Table 2:** Focus group participant characteristics

Characteristic	District A (*N* = 6)	District B (*N* = 8)	District C (*N* = 7)	District D (*N* = 8)	All (*N* = 29)
Age					
20–29 years	0	1	0	1	2 (6.9%)
30–39 years	0	1	1	1	3 (10.3%)
40–49 years	5	3	4	2	14 (48.3%)
50 years and older	1	3	2	4	10 (34.5%)
**Gender**					
Male	5	3	3	3	14 (48.3%)
Female	1	5	4	5	15 (51.7%)
**Professional Category**					
Medical Practitioner– Specialist	1	0	1	1	3 (10.3%)
Medical Practitioner	3	2	2	2	9 (31.0%)
Registered Nurse	2	6	4	5	17 (58.6%)
**Current Position**					
Clinical	3	4	4	5	16 (55.2%)
Management	1	3	2	3	9 (31.0%)
Mixed role	2	1	1	0	4 (13.8%)
**Work Setting**					
District office	1	2	1	2	6 (20.7%)
Hospital	2	3	1	3	9 (31.0%)
CHC/Clinic	2	3	4	3	12 (41.4%)
Two or more of the above work settings	1	0	1	0	2 (6.9%)

### Themes and subthemes

Four themes with multiple subthemes emerged (Table 3).

**Table 3:** Themes and subthemes

Themes	Subthemes
Mental illness is not going away	Mental illness is on the rise
	A surge of substance-related presentations
	A broad spectrum of disorders and presentations
	Patients are getting younger
	High rates of relapse and readmissions
	Social determinants as drivers of mental illness
The health system cannot cope with mental illness	Mental health is not prioritised
	A shortage of mental health units and beds
	Deficiencies at primary health care level
	Human resources for mental health constraints
Clinical associates could be part of the remedy	Clinical associates’ current roles are limited in mental health
	Past experience of other disciplines suggests their potential usefulness
	Constraints and barriers in fulfilling a role in mental health
	Potential roles for clinical associates in mental health and the benefits in utilising them
Specialised clinical associates could help mend the mental health system	Specialisation will allow clinical associates to add value
	Specialisation necessitates review of employment and related policies
	Specialisation necessitates clarification of roles of specialist clinical associates
	Specialisation should be part of the career ladder

### Theme 1. Mental illness is not going away

#### Subtheme 1.1. Mental illness is on the rise

Participants reported that mental illness was a substantial problem in all of their districts.Two of the psychiatric nurse participants (Nurse 1 & Nurse 3, District C) in one of the districts mentioned the overwhelming number of mental health patients per day seen at their facilities. A hospital-based doctor (Medical doctor 1, District D) reported patients in the District D having to spend time in casualty waiting for beds to become available due to the large number of mental health admissions. The numbers of mental health cases were on the rise in their districts, which may be partly due to increased awareness by the public on the one hand. On the other hand, even though the numbers are on the rise, only a small proportion of those with mental health issues are being identified: *“we are aware it is a huge problem but we are really only managing the tip of the iceberg.”* (Medical doctor 2, District D).

#### Subtheme 1.2. A surge of substance-related presentations

The most common mental health presentations were substance-related with participants noting *“a surge in the substance-induced psychiatric disorders”* (Nurse 3, District C) and “*the soaring surge of drug addiction that is taking place in the townships*” (CHC-based manager 2, District C). Substance-related presentations included psychosis and aggression due to use of alcohol, cannabis and *nyaope*. The increased access to some substances due to some being “*legalised”* (Nurse 3, District C) was mentioned as a possible reason for the rise in substance-related presentations. An apparent increase in substance-related presentations in females was a cause for concern.

#### Subtheme 1.3. A broad spectrum of disorders and presentations

Depression was a common presentation at PHC level, but patients with aggression, parasuicide, anxiety (including generalised anxiety disorder), schizophrenia, bipolar disorder, and dementia were also seen. Identification was not always straightforward as “*in the clinics some of the people they hide behind physical diseases*,* they don’t want to come out that they’re having a mental challenge because of the stigma and discrimination*”. (Nurse 1, District B) Many patients end up at hospital due to late presentation because of stigma or a lack of awareness. Participants were seeing patients with the same conditions such as depression, anxiety, schizophrenia, and bipolar disorder in the hospitals, with the notable addition of HIV-associated psychosis:

“*And then in a hospital setting we are still seeing a large number of patients who present with RVD*,* HIV associated psychosis…psychosis being the first manifestation of retroviral disease*,* so they’re actually diagnosed for the first time upon that presentation*,* which you know*,* it speaks to our screening processes for HIV as well. And those patients actually are quite difficult to treat because you have the psychosis and then you need the patient to have insight in order to start ARVs. So*,* it becomes a huge challenge for us in the hospital setting. ”* (Medical specialist 1, District C ).

#### Subtheme 1.4. Patients are getting younger

A concern raised was the increase in younger patients who present with mental health issues. Participants were seeing children present with attention deficit hyperactivity disorder, learning difficulties, behavioural disorders, depression, anxiety, and some that are suicidal. High levels of anxiety and depression were noted in the student population. These presentations sometimes included psychotic features and parasuicide. One participant (Medical doctor 2, District A) estimated that 80% of the mental health patients they were seeing at their hospital outpatients’ department were between the ages of 15 and 30 years. The increase in presentations amongst adolescents and young adults was attributed to substance use: “*So*,* most of the trend that is up and up is amongst youth who are using drugs and they are aggressive and you don’t know whether it’s psychosis*,* or it’s just plain thuggery.”* (Medical doctor 1, District A).

#### Subtheme 1.5. High rates of relapse and readmissions

The high rates of relapse among mental health patients and the need for readmission was a common experience. The reasons provided for this included patient defaulting treatment, the lack of rehabilitation facilities and beds for substance-related problems, and lack of appropriate social support. A few explanations were offered for the ‘revolving door syndrome’:

“…*despite the patient requiring a higher level of care*,* they’re not actually getting assessed and managed by a person that has any additional qualification. So*,* it contributes to that revolving door syndrome where not much has changed when the patient is discharged and then down referred to the CHC”* (Medical specialist 1, District C).

*“…as a hospital we are doing this much*,* and then we are sending the same patient to the same environment that one was in. Meaning that we are just going round circle.”* (Hospital-based manager, District D).

“…*a revolving door that you come*,* you’re seen*,* go back to the situation*,* there’s no rehabilitation*,* there’s nothing. And then it becomes a huge problem because we can’t manage and we can’t contain it because of that neglect. ”* (District-based manager, District D).

#### Subtheme 1.6. Social determinants as drivers of mental illness

The socioeconomic circumstances of individuals and communities was driving the high rates of mental illness. In particular, high unemployment in their districts was mentioned as the underlying reason for acute stress and substance use. It was also thought that unemployment was driving the youth to join gangs where they have to comply with the substance use norms of the group. The fragmentation and breakdown of community support structures was also playing a role in the increase in mental health issues particularly those related to substance use. Gender-based violence was linked to the increase in mental illness among women. The stress of women being the main breadwinner in many families may also be contributing to the rise in mental illness in females:

*“So*,* in fact*,* the cause of most of this cases to increase amongst women is that the women now are the ones who are breadwinners in their families. They are the ones who take responsibility of the kids and ensure that the children end up eating. Where the husband or their partners they don’t care. They concentrate on alcohol and other stuff; they use their money there. And that women… have the stress of managing everything at the same time*” (District-based manager, District A).

### Theme 2. The health system cannot cope with mental illness

#### Subtheme 2.1. Mental health is not prioritised

The lack of prioritisation of mental health by the authorities with respect to financial and human resources was highlighted by participants. It is still considered the “*step-child of health*” (Nurse 3, District B). One participant noted that their provincial budget for mental health was so meagre that they could not do the necessary training to upskill staff or hire specialised staff. The consequences of the lack of long-term lack of prioritisation and planning was noted: “*But because of the neglect from the health department itself*,* because we never seriously*,* you know*,* looked at mental health as a challenge and as a problem*,* and now we are reaping the fruits of that neglect…”* (District-based manager, District D).

The failure to implement the Mental Health Policy Framework For South Africa And Strategic Plan 2014–2020 [29] was mentioned:

*“…if you take a closer look at the framework*,* which is a national theme*,* to me it has failed and if that has failed*,* I mean*,* everything going downwards*,* because it talks about resources*,* it talks about capacitation*,* it talks about all those things.”* (Nurse 3, District B).

#### Subtheme 2.2. A shortage of mental health units and beds

A lack of facilities to deal with mental health patients was raised as a concern. In one of the districts, there is only one designated mental health unit in the entire province and in two districts, there is such a shortage of inpatient beds, that mental health patients end up spending long periods in casualty:

*“…acute presentations that are being admitted for 72 hours in a hospital setting*,* where we frequently have a shortage of beds; and so patients have to often spend their 72 hours of observation in a casualty…which is not ideal”* (Medical specialist, District C).

The shortage of beds at tertiary level result in delays in referring patients requiring higher levels of specialised care, and families often do not want to accept patients back post-discharge which further exacerbates the shortage. The rise in adolescent presentations added a further challenge as “*we didn’t really cater for that…we don’t have beds for adolescents*,* then now you suffer because you must close one ward for one person for a long time.* ” (District-based manager, District D) Given the high rates of substance-related presentations in all of the districts, the lack of rehabilitation facilities and beds is a major challenge:

*“…those who are willing maybe to go for rehab we’re having challenges because the list is too long. They end up going back to the community. And if they are willing to quit*,* they usually relapse. And the cycle goes on and on and on”* (Medical specialist, District A).

#### Subtheme 2.3. Deficiencies at primary health care level

The system-wide dysfunction is obvious: “*If you look at the district system now of mental health*,* it is also in disarray. The whole mental health management in the primary health setting and in the health system is not functioning*.” (CHC-based manager 2, District C) A shortage of psychiatrists has resulted in the lack of specialist outreach to CHCs. There is an absence of mental health specialist teams covering community services. The limited list of psychotropic medication that can be prescribed at PHC level and the stockouts hamper service delivery. Filling scripts for chronic mental health patients at PHC level was a challenge because of the lack of medical officers who are required to sign the prescriptions. Transferring patients, particularly those that are aggressive, from PHC level to hospitals is hampered due to the lack of appropriate means of transport. The lack of data being collected at PHC level with respect to mental health has a detrimental effect as the scale of the problem cannot be quantified.

The need for screening and early detection of mental health issues was acknowledged but there *“isn’t sufficient effort to try and detect mental illness at the primary healthcare level”* (CHC-based manager 1, District C). One explanation was that there is little point to screen at PHC level if they cannot provide treatment or care. The lack of screening tools for child and adolescent patients is a gap.

The system dysfunction extends to the overlooked potential role of community health workers in community-based prevention and early detection of mental illness as the dominant approach is still curative and hospi-centric:

*“Which then is compounded by the fact that the approach is very much a hospi-centric approach. In other words*,* we wait in hospital for people to be sick enough to get to somebody who has the level of skill required to make the correct diagnosis and then link them to care and put them on the correct treatment”* (CHC-based manager 1, District C).

#### Subtheme 2.4. Human resources for mental health constraints

The care and treatment of those with mental illness needs appropriately skilled personnel, who are in short supply: “*we end up seeing more people because of the shortage of human resources. Actually*,* I can say*,* the shortage of well-equipped personnel. Because they can be there*,* but then not well-equipped to assist in the problem at hand.* ” (Nurse 1, District C) Concerns were raised regarding the ability of non-specialist health professionals to adequately address mental illness: “*we have nurses that are dealing with those*,* and you find that others are not even trained. They don’t have the skill to do that.”* (Hospital-based manager, District D) The ability of medical officers to manage mental health patients was called into question: *“So definitely our doctors lack adequate psychiatric knowledge and training to be able to be the first point of contact…”* (Medical specialist 1, District C) There was a lack of training opportunities for non-specialists. It was felt that nurses and medical officers needed ongoing refresher training in mental health and for medical officers to complete the Diploma in Mental Health. One of the training gaps identified among medical officers was on the documentation needed to be completed in terms of the Mental Health Act, 2002. In one district, the medical officers at PHC level are generally those doing their one year of compulsory community service which brings its own complications: “*every other day it’s the community service doctors. They don’t have any knowledge at all with regards to psych*,* so that leaves my colleague and myself with like 22 years of psych experience and advance psych training to assist them*”. (Nurse 3, District C) One participant bemoaned the fact that 12 months is spent transferring skills to these doctors who then leave at the end of their community service.

The universal concern is the shortage of key members of the specialised mental health workforce with one district reporting that they do not have any psychiatrists while two others had access to a single psychiatrist only. The shortage of psychiatrists also meant there was a lack of capacity in the districts to provide in-service training to non-specialists. Psychologists are also in short supply with the result that when the waiting time is too long for an appointment, patients never return. The shortage extends to the lack of specialised registered nurses and social workers which adds to the inefficiencies in the system. For example, a psychiatric nurse based at a CHC (Nurse 1, District C) described how they sometimes have to send patients home without being assessed and ask that that they come back the following day because they are so overwhelmed. The lack of multidisciplinary teams means that patients might only see a single practitioner who cannot address all the patient’s needs: “*we don’t have holistic teams…so maybe I might be prescribing but there’s huge other issues*,* which I think we often at our level*,* and it sounds like every level*,* feel overwhelmed actually.*” (Medical doctor 2, District D).

### Theme 3. Clinical associates could be part of the remedy

#### Subtheme 3.1. Clinical associates’ current roles are limited in mental health

Although clinical associates are in a variety of settings in the different districts, none of these include mental health settings beyond outpatient departments and emergency departments. Those at PHC clinics may be seeing mental health patients as part of the general patient population but there was a concern that they may be missing a number of these patients. There was a reluctance to place clinical associates in a specialised mental health setting: “*So*,* for us*,* to be honest*,* in terms of mental health per se*,* we’ve shied away actually to put a clinical associate into that clinical discipline.”* (Hospital-based manager, District B).

#### Subtheme 3.2. Past experience of other disciplines suggests their potential usefulness

Participants acknowledged the added value that clinical associates bring to the health system and were complimentary of the work that they do in other areas: “*very efficient when it comes to treating patients and also picking up diagnoses. I wouldn’t say I’ve had any problems.”* (Medical doctor 1, District D). Past experience of their added value was extended to their potential role in mental health: “*you know they are not the same but some of them have performed incredibly well and have shown that they can cope incredibly well in other situations. And I have no doubt that they could do the same here in psychiatry if they were to be given the adequate training”* (CHC-based manager 1, District C), and: “*my opinion is that they can work in any department as long as there’s supervision. So*,* it’s not only about mental health*,* even general medicine*,* if there’s no supervision there will be mistakes.”* (Medical Doctor 1, District A).

Retaining clinical associates in the public sector is a key success factor as experience counts:

“*What happens when they land*,* I would say when they first come from the university*,* they are a little bit on shaky ground*,* so most of them they’re still behind. But with time I think we’ve had others that have been with us for five/six years*,* you can say that actually these function*,* they’re usually much better than most of the doctors actually that are there.”* (Hospital-based manager 1, District B).

*“Because like the rest of us*,* when you start working your training hasn’t prepared you for everything and as long as you are able to learn…and also a huge thing in my personal experience is having a support of specialists or people that you can phone. So*,* it’s the ability to recognise things and then to seek help when you’re unable to manage it and learn from that.”* (Medical doctor 2, District D).

#### Subtheme 3.3. Constraints and barriers in fulfilling a role in mental health

The limitation of their scope of practice restricts their potential usefulness in mental health: *“the issue of limitation in terms of their scope of practice. That’s where we are encountering the problem.”* (District-based manager 1, District B) It was noted that circumstances on the ground (e.g. shortage of medical officers) has dictated that some clinical associates *“have gone beyond their scope of practice.”* (Hospital-based manager 1, District B) A significant limitation and cause of inefficiency is that unlike some nurse practitioners, clinical associates need their prescriptions to be countersigned by a medical doctor: “*they’re just clipping their wings*,* the fact that they cannot prescribe.”* (Clinic-based manager 1, District B) As one of the motivations for the creation of this cadre was to supplement the shortage of medical doctors in the public sector, a rational solution would be to amend the legislation: *“although the law says no dispensing*,* let the law be amended in such a way they do the dispensing course and then if they qualify then you allow them actually to dispense.”* (Hospital-based manager 1, District B). The example and positive outcomes of registered nurses being allowed to prescribe and dispense medication at PHC level was thought to be similar enough to argue for the change in legislation. Another area of legislation that needs to be changed is the Mental Health Care Act, 2002 [30] as mental health care users are “*supposed to be examined by the medical doctor. The Act doesn’t… cater for clinical associates to examine the patient.”* (District-based manager 1, District A).

The restriction of supervision for clinical associates poses a particular challenge at PHC level as there are many in PHC clinics without medical officers. The envisaged benefit of supplementing the shortage of medical doctors is hampered as *“sometimes* [it] *is more work*,* because now it’s two people seeing the same person*,* versus one person seeing them.”* (Medical specialist 1, District C). Busy medical officers have to supervise clinical associates in addition to managing patients, which is a risk of as clinical associates might not receive appropriate supervision leading to errors.

Currently, clinical associates are not equipped *“to conduct the proper mental health assessment. They would not even be able to give a provisional formulation of a diagnosis.*” (Nurse 3, District B) The two to four weeks of training in mental health in their undergraduate curriculum was viewed as inadequate on its own. While strengthening the undergraduate training programme was one option, it could also be addressed after they qualify through working in a supportive environment and additional training:

“…*with the capacitation as we always do when people come…you are never ready for all the things. But when people come*,* we do take them through and there are courses that we send people to*,* then we can prepare them and refocus them so that they can manage and they will be able to manage*,* and with additional training and refocusing them upon mental health.”* (District-based manager 1, District D).

The competencies required to manage mental health patients take years to develop as these patients tend to be complex and differences in diagnoses are often quite subtle. Potential incorrect and missed diagnoses by clinical associates could have medico-legal implications particularly with respect to complex cases such as suicidal patients and children. It was noted that children were particularly challenging to assess and diagnose with respect to mental health. In addition, there are those who apply for disability grants for mental health issues and whose assessment requires substantive competence. Given clinical associates are a relatively new health cadre in SA, their acceptance by mental health patients and their family members is a potential barrier: “*And from the public point of view*,* how receptive is the public going to be and how comfortable are they going to be that my ill relative or child is going to be assessed and diagnosed by clinical associates.*” (Medical specialist 1, District C).

#### Subtheme 3.4. Potential roles for clinical associates in mental health and the benefits in utilising them

Clinical associates could be a welcome addition to the multidisciplinary team despite their current shortcomings as they can assist in addressing the lack of mental health services:

*“I think so we could allow them to focus on mental health and help us in that space*,* because as you can see*,* we are really suffering when it comes to mental health*,* and probably because the service*,* as I said*,* was never planned*,* was neglected. So now in refocusing on mental health*,* like we are primary healthcare reengineering*,* I’m sure we are mental health reengineering*,* so we could make a platform that includes a clinical associate especially in our district because we don’t have the services at all. We are failing as far as mental health is concerned.”* (District-based manager 1, District D).

The role definition at PHC level would be to screen, make an early diagnosis and manage patients who are referred back to PHC after specialist care. They would, therefore, have a role to play prior to referral, once the patient is down referred, and with the “linkages”. It was noted that at PHC level, mental health patients are integrated into the general patient workload to avoid stigma so clinical associates should be able diagnose and manage a wide range of common physical and mental health conditions and presentations. At hospital level, they could be used in casualty to do the initial assessment of patients presenting with mental health issues as well as work in 72-hour observation units as many of the tasks may not require extensive mental health knowledge and skills: “*you fill the form*,* you have the blood to be taken*,* you manage*,* you rule out any general medical condition*,* and then you transfer if the patient need to be transferred*,* you see. I think in our institution that is feasible.”* (Medical specialist 1, District A) Clinical associates could be involved in mental health outreach and education in communities as well as monitoring adherence to psychotropic medication as part of home visits.

The lessons learnt in scaling up treatment for HIV is analogous with the use of clinical associates in the provision of mental health services:

*“So*,* we are never going to be able to create sufficient psychiatrists to be able to match the demand that we are facing at the moment… And I make this analogy deliberately that says that with ARV medication now the number of people that are on ARV medication is huge*,* and it is not because we’ve got sufficient specialists*,* it is because there was a transfer of skills to the nursing personnel to ensure that we are able to reach the large numbers of people. If we agree that the number of mental health cases has increased then we do need to think beyond just training more specialists.”* (CHC-based manager 1, District C).

### Theme 4. Specialised clinical associates could help mend the mental health system

#### Subtheme 4.1. Specialisation will allow clinical associates to add value

Postgraduate training in mental health for clinical associates was viewed as a natural solution to address the complexities of mental health: “*It will help a lot if they have that mental health training of which to me it will be something like… a diploma. So*,* if they do have that capacitation*,* I think they will also assist us a lot.”* (District-based manager 1, District B) Additional training would help to close gaps in their skills, reduce the chances of mistakes, increase confidence of supervisors in their abilities, and make them more useful. An additional year of training was proposed as one of the ways to ensure competence to work in mental health:

*“…how about they qualify*,* then maybe you plan an additional one year in psychiatry*,* then put them there*,* rather than sending them with only a four weeks training or exposure. How about they are clinical associates then they have additional one year*,* at least*,* maybe two…more of an assistant. And then after that they can be deployed to wherever to assist. I believe that one year can be better.”* (Nurse 1, District C).

The creation of specialist clinical associates would address the reality of having medical officers without the appropriate training. These clinical associates would be more equipped than community service doctors who are currently expected to assess and initiate treatment for mental health patients at PHC level. Building on the lessons learnt in the HIV pandemic, it was clinical associates with advanced training in mental health could have a substantial impact:

*“We realised we didn’t have enough medically trained people to be able to tackle the vast numbers of people that needed ARV medication. And training nurses*,* there’s NIMART training with nurses*,* and trying to bring them in*,* I believe has made a massive impact to ensure that people are…after diagnosis*,* are linked to care*,* and are maintained on that.”* (CHC-based manager 1, District C).

These clinical associates could potentially also add value to multidisciplinary teams where these exist. Not all participants agreed, as it might be more efficient to focus on the existing cadres rather than pursuing advanced training for clinical associates. One participant expressed the view that additional training for clinical associates may still not be adequate given the complexity of mental health patients and could potentially give a false sense of security: “*And if we are going to be going to that safety net that this person’s now had additional training so they should be confident to be on their own*,* we’re going to miss these cases*.” (Medical specialist 1, District C).

The benefit of providing a career path in mental health for clinical associates particularly for those with a stated interest in the field could lead to the creation of a sustainable mental health workforce:

“*you’ve now completed your degree*,* you have a particular interest in psychiatry or mental health*,* then you would spend that requisite time required*,* and then you come out of there*,* and nobody would really want to do that unless they wanted to stay in that particular discipline for long enough.”* (CHC-based manager 1, District C).

#### Subtheme 4.2. Specialisation necessitates review of employment and related policies

The introduction of a specialisation for clinical associates would require a number of regulatory and operational adjustments. Their advanced qualification in mental health would need to be recognised by the Health Professions Council of South Africa and these specialised clinical associates would need to be included in the organograms of districts, and posts would need to be budgeted for. The scope of practice of such a specialised clinical associate would need to be clarified as:

“*I will rather create that post for a medical officer that is going to prescribe*,* because remember even if they do the postgrad diploma they still need a medical officer to countersign their prescriptions. So*,* to me*,* to cut the costs I’d rather have the medical officer and the psychiatric nurse managing my services in primary healthcare because that is our focus now.”* (District-based manager 1, District D).

The role of clinical associates is not clear in the proposed National Health Insurance (NHI) policy and if sufficient medical doctors would be contracted to provide PHC services, clinical associates might be “*redundant in primary healthcare*,* because you’re going to have a doctor that is going to deal with all of that*”. (District-based manager 1, District D)

#### Subtheme 4.3. Specialisation necessitates clarification of roles of specialist clinical associates

Specialist clinical associates could still have a role to play at PHC level. They could then refer where necessary to the secondary level for the treatment plan and the patient could then be referred back to the community. This strategy would reduce pressure on the secondary level: “*And then that of course would also help with the influx of patients coming from the community to the secondary level of care.”* (Nurse 3, District B) They could also review treatment of mental health patients on periodic basis (three or six monthly) as there is usually a “crisis” when there are no medical officers around to do this: *“But if they are trained*,* they are skilled enough*,* they will be able just to assess the patient if ever there is a need to change medication they will be in that position in consultation with psychiatrists”* (District-based manager 1, District B). The limitations regarding prescribing would need to be addressed as they: *“would be more of value further that they’re able to handle a patient up until the end where actually they dispense or refer as necessary”* (Hospital-based manager 1, District B).

There was not universal acceptance of the value of specialist clinical associates in mental health at PHC level:

“…*we are mainstreaming mental care into the whole primary care setup to see that person in totality with everything and to fit in the society. We don’t want to run mental health clinics in the PHC firstly to discriminate…we want them to believe you’re normal as much as possible to be able to sit in the line with others. So*,* this model now of a clinical associate which cannot see and prescribe and do everything in totality is going to be a shortfall.”* (District-based manager 2, District D).

It was felt that an “*all-rounder*” rather than a specialist is required at PHC level. There was a concern that mental health patients might be stigmatised “*when you are seen by that room”* (District-based manager 1, District D).

Their envisaged role at hospital level included screening and assessing patients in casualty, follow up of patients in outpatient departments and identifying contraindications and side effects related to medication if they were allowed to prescribe. There was one argument made for a potential role for them in specialised psychiatric units.

#### Subtheme 4.4. Specialisation should be part of the career ladder

The lack of career pathing for clinical associates had not gone unnoticed by participants: “*I’m of the opinion that I think we have done injustice by developing the clinical associate category of workers. Here I’m talking about in terms of the career path being I think they are stuck in limbo.”* (Hospital-based manager 1, District B) Participants who had worked with clinical associates were aware of clinical associates’ frustration regarding this lack of opportunities and the absence of career pathing. This resulted in a number of them wanting to leave the profession and study medicine, and specialisation would be a potential solution: “…*if there was a way to channel them to their area of interest and have some kind of career progression it might help*,* but at the moment it’s a dead-end destination here and it does present a bit of a problem.”* (CHC-based manager, District C).

## Discussion

Our study aimed to explore the views of health managers and clinicians in four districts of SA on mental illness in their districts and mental health presentations to health services, the challenges faced in providing services, and their attitudes towards mental health task sharing involving clinical associates.

The view expressed by participants that mental health is a substantial problem in their districts is not surprising as epidemiological data suggests high prevalence rates of mental illness in SA [31–33]. The magnitude of the problem is supported by studies that have screened for one or more mental health disorders in healthcare settings where large proportions of participants (20% or higher) have screened positive for at least one mental health disorder [34–39]. The high rates of substance-related presentations were seen in all four districts. Nationally representative data has found a lifetime and 12-month prevalence of substance use disorder in SA of 13.3% and 5.8% respectively [31]. Kagee et al. [36] found a prevalence for just alcohol use disorder of 19.8% amongst individuals presenting for HIV testing in the Western Cape. Substance use was linked to the increase in mental health presentations being seen in younger people which is confirmed by a meta-analysis of substance use among adolescents in sub-Saharan Africa which reported a prevalence of just under 37% for the use of any substance among South Africans [40]. High rates of substance use have been reported for secondary school learners and university students in SA [41, 42]. Depression was also a common presentation particularly at PHC facilities as one would expect based on studies that have reported on depression in SA [31–37, 39].Participants felt that unemployment was the underlying reason for the rise in mental health presentations they were seeing. There is evidence for the link between unemployment and poor mental health [43–45]. SA has one of the highest unemployment rates in the world and was recorded at 32.9% in early 2023 [46].

According to participants, the high rates of relapses and readmissions is a major contributor to the numbers of mental health patients having to be managed at hospital level. This pattern is a common experience throughout the country with Docrat et al. [2] reporting an average overall readmission rate of 24.2% for mental health patients within three months of previous discharge. They estimated that for the 2016/17 financial year, this cost 112.6 million US Dollars and was responsible for 18.2% of total mental health expenditure [2]. We found deficiencies at PHC level in our study which may be contributing to the lack of continuity of care once patients are discharged. Sorsdahl et al. [4] have noted the importance of strengthening referral pathways when patients are discharged to primary care and community level to ensure continuity of care.

Although we were interested specifically in human resources for health challenges related to mental health, other mental health systems challenges emerged from the focus groups. Participants felt that mental health was seen to have been historically neglected in SA and is still not being prioritised. Despite progressive mental health legislation and policy in SA, this neglect of mental health is borne out by the relative lack of expenditure on mental health and the failure of policy implementation [2, 4]. Bird et al. [47] identified several reasons for the low priority given to mental health in SA viz. the prevalence and severity of the problem not being understood, lack of knowledge regarding appropriate interventions, socio-cultural beliefs on aetiology and treatment, a lack of funding, a lack of advocacy, and stigma. The lack of designated space or specialised units for 72-hour observation of mental health patients admitted under the Mental Health Care Act, 2002 has long been recognised as an issue [4, 48, 49] and our findings suggest that this is an ongoing problem with some patients having to spend their entire 72 h of observation in casualty departments. The delays in transferring patients who require it to higher levels of care due to shortages of beds at referral facilities has also been previously documented [4]. The lack of rehabilitation facilities for substance use disorders was a major concern among our participants given the large number of patients being seen with substance-related issues. The lack of availability of beds for the growing number of adolescent patients found in our study provides evidence to support Sorsdahl et al. [4] that there is a shortage of inpatient facilities for adolescents necessitating the use of adult wards. Given the increase in the number of child and adolescent patients with mental health issues, the lack of appropriate screening tools is a gap that needs to be addressed. The approach to mental health in SA remains hospi-centric one [2, 4]. Mental health care at PHC level accounted for only 7.9% of the total cost of outpatient and inpatient mental health services in the 2016/17 financial year [2]. The lack of mental health data being collected at PHC level is also a barrier in advocating for more resources.

Predictably, mental health workforce shortages were a major challenge reported by all four districts particularly the lack of psychiatrists and psychologists. The ability of non-specialists such as generalist registered nurses and medical officers to manage mental health patients was questioned. When PHC facilities do have doctors, it is often community service medical officers with limited mental health training and experience who manage mental health patients, and when there are no doctors in these settings, there is no one to prescribe psychotropic medication [4]. Our study highlights the possible contribution of clinical associates to address some of the deficiencies in mental health service provision. Some of the envisaged roles have previously been suggested in research by Moodley et al. [19] including working in in emergency settings (casualty) and 72-hour observation units, assessing adherence to medication as part of home visits, and mental health promotion activities in communities [19]. In addition, there is a role for them in the co-ordination of care role at PHC level that includes doing the initial assessment, referring the patient for initial diagnosis and treatment and then managing the patient after the patient is down referred [19].

Clinical associates were not being utilised in mental health service provision in any of the districts in this study. Although participants did not have first-hand knowledge of how clinical associates would perform in this discipline, participants had positive views on how they could contribute based on their contribution in other settings. This finding is consistent with other studies which noted their levels of professionalism and skills, efficiencies in patient management such as reduced patient waiting times and increased patient satisfaction, reduced workload for medical doctors, and improved access to healthcare [50, 51].

Several legal and operational changes need to be considered for full utilisation of clinical associates in the provision of mental health services. The limitation in their scope of practice particularly regarding supervision and prescribing would require revision. The requirement that prescriptions be countersigned by the supervising medical doctor has been previously flagged as a hindrance to providing greater efficiency [51]. A further complication with respect to using clinical associates (and registered nurses) in mental health service provision is that they are only permitted to prescribe up to Schedule 4 medications while psychoactive medicines (e.g. anti-depressants) are Schedule 5 [17, 52]. Concerns related to the supervision regimen have been raised previously, especially in the light of the shortage of doctors who have to supervise them [15, 50, 51]. Any changes to the supervision requirement would need to take any training deficiencies and medico-legal concerns into account [51, 53]. While the majority of participants were not familiar with the training programmes for clinical associates, there was a view that the current training on mental health was probably inadequate based on the time allocated to it. Moodley et al. [18] have made a number of recommendations to strengthen undergraduate mental health training including focusing on conditions they are likely to see in practice and dedicated time in a mental health unit. It has also been suggested that short courses in mental health be used to close the gaps for those that have already qualified and want to work in this field [19].

Advanced training for clinical associates in mental health (e.g. through a postgraduate diploma) was generally supported by participants. The reasons provided were similar to the findings by Moodley et al. [19] among those involved in clinical associate training programmes which broadly categorised the reasons for supporting such an option as those related to strengthening the health system (e.g. improving access to mental health services), strengthening the clinical associate profession (e.g. creating career pathing), and individual reasons (e.g. interest in the discipline). The training of clinical associates in mental health was compared with the training of registered nurses on antiretroviral therapy to address the HIV pandemic. Sorsdahl et al. [4] in reflecting on task-sharing in mental health mentions HIV as an example of how task-sharing has been successfully implemented in the South African context. The structural barriers that would need to be overcome to utilise clinical associates with advanced training (specialisation) in mental health include the recognition of the advanced qualification by the Health Professions Council of South Africa, changes in scope of practice (particularly regarding the need for supervision and prescription rights), changes in the Mental Health Care Act 2002 [30], the creation of posts, and the funding of posts.

It was not clear in this study how the roles for those clinical associates with advanced training in mental health would differ sufficiently from those with undergraduate training only. This delineation would need to be clearly defined prior to developing any future postgraduate qualification in mental health. Results of a Delphi panel of family physicians and psychiatrists reported by Moodley et al. [54] provides some guidance on how the roles of clinical associates with a postgraduate mental health qualification may differ from those with mental training as part of their undergraduate qualification only.

This study has highlighted the need for further public mental health research generally as well as additional studies to explore the role of clinical associates in mental health task-sharing specifically. Nationally representative survey data would be useful to confirm the views expressed in our focus groups that mental illness prevalence is increasing and to provide insight on who is being affected. Research on health system and individual factors contributing to high rates of readmission and the evaluation of strategies to reduce readmissions are required. Screening at primary care level requires further research that helps to identify the target groups to screen, the mental illnesses to screen for, and the appropriate screening tools to use. The utilisation of clinical associates in mental health services can be further evaluated through piloting task- sharing interventions and implementation studies.

### Limitations

The number of focus groups conducted was pre-determined due to the complexities of logistical arrangements and was not based on data saturation. It is possible that additional focus groups may have yielded further information. The focus of the study was on the health provider (doctors and nurses) and health manager perspective. We used purposive sampling to ensure each focus group consisted of a diverse combination of participants who would be able to provide as rich information as possible based on the study objectives. As this is a non-probability technique, the views expressed by the focus groups may not be broadly representative of health providers and managers in each of the selected districts. Clinical associates were not included in the focus groups. While their views may have provided additional insights, it was felt that their presence would inhibit the responses of other members of the focus groups particularly those holding views that may have been critical of clinical associates’ work or unsupportive of their involvement in mental health service provision. This study was, however, part of a larger research project which included both qualitative and quantitative components that included clinical associates [18–20, 55]. Individuals with lived experience were also not included and this may be an avenue of future research as concerns were raised regarding the potential acceptance of the relatively new clinical associate cadre by mental health patients and their families.

The focus groups consisted of a mix of registered nurses, doctors, and managers at differing levels of seniority. It is possible that some participants may not have felt comfortable expressing themselves freely in focus groups in the presence of senior colleagues particularly if they had differing views. While the districts selected all employed clinical associates, the numbers employed were generally small and they were mainly hospital-based which meant that some participants had not worked with clinical associates directly and were not able to speak from first-hand experience. The focus group discussions were conducted in English only. While all participants appeared comfortable communicating in English, it is possible some may have interacted more readily in their first language.

## Conclusion

South Africa’s public health sector faces significant challenges in providing mental health services with human resources for mental health being a critical issue. These deficient resources are shortages of the specialised mental health workforce as well as non-specialists inadequately prepared to deliver mental health services. Clinical associates may have a potential role to play in addressing some of the human resource constraints in mental health with a strengthening of their undergraduate training and an advanced training offering in mental health such as a postgraduate diploma. In order to fully realise their potential in mental health service provision, structural barriers including their scope of practice, regulatory issues, and the creation and funding of posts will need to be addressed.

## Supplementary Information

Supplementary Material 1.

## Data Availability

The datasets used and analysed during the current study are available from the corresponding author on reasonable request.

## Abbreviations

CHC: Community health centre
PHC: Primary health care
SA: South Africa
