# Supplementary material for: Utilising clinical associates to address mental health service provision challenges in South Africa: the views of healthcare managers and providers
Source: BMC Health Serv Res. 2025 Aug 18;25:1090. doi: 10.1186/s12913-025-13300-9 (PMC12359855; doi:10.1186/s12913-025-13300-9)
Supplement: Supplementary file 1 — Supplementary Material 1. [file 12913_2025_13300_MOESM1_ESM.pdf]

Focus Group Interview Guide

Date:

Time of Focus Group:

District:

Interviewer:

Number of participants:

1. How big a problem is mental illness in your district?
   1a. What are the common illnesses seen?

2. Do you think there are sufficient human resources to deal with mental illnesses in your district?
   2a. Are they adequately trained?

3. Where are clinical associates currently based in your district?
   3a. What work are they currently doing?

4. What are your views on whether clinical associates are equipped to manage mental health issues?

5. Are you open to clinical associates currently in your district playing a greater role in managing mental illness in your district?
   5a. What work in mental health do you think clinical associates can do (if 4 = yes) or why not (if 4 = no)?

6. Are you open to posts being created for clinical associates to work specifically on mental health in the district?

7. What do you think would be the health system challenges to adopting mental health task sharing involving clinical associates?
